# Social wellbeing, values, and identity among Caiçara small-scale fishers in southeastern Brazil

**DOI:** 10.1007/s40152-023-00322-4

**Published:** 2023-08-12

**Authors:** Marta C. F. Leite, Derek Stephen Johnson, Helen Ross, Cristiana Simão Seixas

**Affiliations:** 1 Natural Resources Institute, University of Manitoba, Winnipeg, Manitoba Canada; 2 Department of Anthropology, University of Manitoba, Winnipeg, Manitoba Canada; 3 School of Agriculture and Food Sciences, The University of Queensland, St Lucia, Queensland Australia; 4 Environmental Studies and Research Center, University of Campinas, Campinas, São Paulo, Brazil

**Keywords:** Social wellbeing, Caiçara, Brazil, Small-scale fisheries, Artisanal fisheries, Identity, Values, Governance, Natural resources management, Livelihood, Ethnography

## Abstract

Although much in the lives of members of the Caiçara small-scale fishing communities of Lázaro and Saco da Ribeira in Ubatuba, southeastern Brazil would suggest hardship, that population expresses a surprising degree of satisfaction with life. In this paper, we use a social wellbeing lens as applied through an ethnographic, mixed methods approach to reflect on this overall sense that lives rooted in small-scale fishing are well worth living despite their many challenges. We see the classic maritime anthropology theme of identity at the heart of meaning and life satisfaction. Identity provides core aspects of how people engage with their realities and anchors values that are reference points in work and social relations. With reference to the relational nuances revealed by the social wellbeing perspective, however, we show that Caiçara and small-scale fishing identities are not monolithic, but reflect gender and other social positions, and personal and familial experiences. These experiences include grappling with the complex effects of economic, social, political, and environmental changes. We conclude by arguing that fisheries policy that seeks to prioritize human wellbeing would benefit by adopting a social wellbeing perspective. Fisheries policy could thereby take into account identity, values, and relational elements of social life that give meaning and a sense of belonging to small-scale fishers, while also recognizing the cross-cutting and often contradictory variations in human experience that arise from social and economic differences. This social fabric of small-scale fishers’ lives shapes their intentions and actions and is thus a necessary complication to the practice of fisheries management that its proponents need to consider.

## Introduction

A core assertion of the maritime anthropology literature of the 1980s and 1990s was that there is a distinctive quality to fisheries that is worthy of study and explanation (Acheson 1981; McGoodwin 1990). The broader social science literature on fisheries has largely implicitly, but occasionally explicitly (see Béné 2003), made the argument that the study of fisheries embodies a microcosm of broader human issues related to institutions, meanings, and relationships. The more recent literature has picked up these points about the uniqueness, but also the broader societal interest of fisheries in its various arguments in support of small-scale fishers and their communities (Jentoft 2020). The literature argues that small-scale fisheries have value not only materially in terms of livelihoods, nutrition, food security, and in contributions of knowledge but also for the intangible and distinctive contribution they make to global human heritage (FAO 2015; Urquhart et al. 2014; Fabinyi and Barclay, 2022).

In recent years, the social wellbeing approach (Gough and McGregor 2007) has been taken up as a framework and methodology to convey the societal importance of small-scale fisheries, to build understanding of the particular qualities of the sector, and to provide insight into the life-worlds of small-scale fisheries and their participants (Coulthard, Johnson, and McGregor 2011; Weeratunge et al. 2014). An early example of the application of the approach was Coulthard’s analysis of how subjective perceptions related to group belonging strongly influenced how different groups adapted to new fishing opportunities (Coulthard 2008). According to the social wellbeing approach, wellbeing perceptions are not fixed, but are rather positional and relational (Johnson 2018). Wellbeing, from this perspective, is about what people value being and doing, as well as how they relate to the environment to which they belong (Millennium Ecosystem Assessment 2005; Armitage et al. 2012; Idrobo 2014).

With reference to small-scale fisheries in Ubatuba (Sao Paulo state, Brazil), we apply the social wellbeing approach in this paper as a lens to showcase the complexity of attachments to fisheries and belonging to place, the tensions in social relations in the face of change, and to explore and test the limits of earlier notions that fisheries foster a distinctive identity, and even culture. We trace how complex relationships among social difference, values, and identity inform understandings and practices of living well. We affirm the importance of social wellbeing as a frame of analysis and suggest it could be a means through which to build empathetic understanding of the contextually specific qualities of small-scale fisheries among those responsible for governing the sector. These empirically grounded and practice-relevant reflections on social wellbeing complement other recent research on identity and wellbeing in Brazil and elsewhere (Idrobo and Johnson 2020; Biswal and Johnson 2023; Belton et al. 2018; Stacey et al. 2018; White 2018). Our study is distinctive, however, in using social wellbeing to reveal the social and economic differences that complicate shared small-scale fisher and Caiçara identities.

In what follows, we first present the social wellbeing approach, then our study site and its people, followed by the diverse research methods used. Subsequently, we lay out our findings in relation to the core theme of the commonalities and contradictions in expressions of identity and wellbeing among small-scale fishers in Ubatuba. We conclude with a return to implications of our findings for the fisheries literature.

## The social wellbeing approach: values, identity, and social relations

The social wellbeing approach, developed by the Wellbeing in Developing Countries Research Group, linked to the University of Bath (UK), provides a holistic and multidimensional approach and a people-centered lens as it seeks to understand people’s objective circumstances, their subjective perceptions of these circumstances, and the relationships that shape how they perceive their quality of life (Gough and McGregor 2007). Armitage et al. (2012: 15) define social wellbeing as “a state of being with others and the natural environment that arises where human needs are met, where individuals and groups can act meaningfully to pursue their goals, and where they are satisfied with their way of life.” The social wellbeing approach builds on several bodies of literature, including Sen’s capabilities approach, the sustainable livelihoods approach, economics of happiness, and gender studies (see: Weeratunge et al. 2014 for more details).

**Fig. 1:**
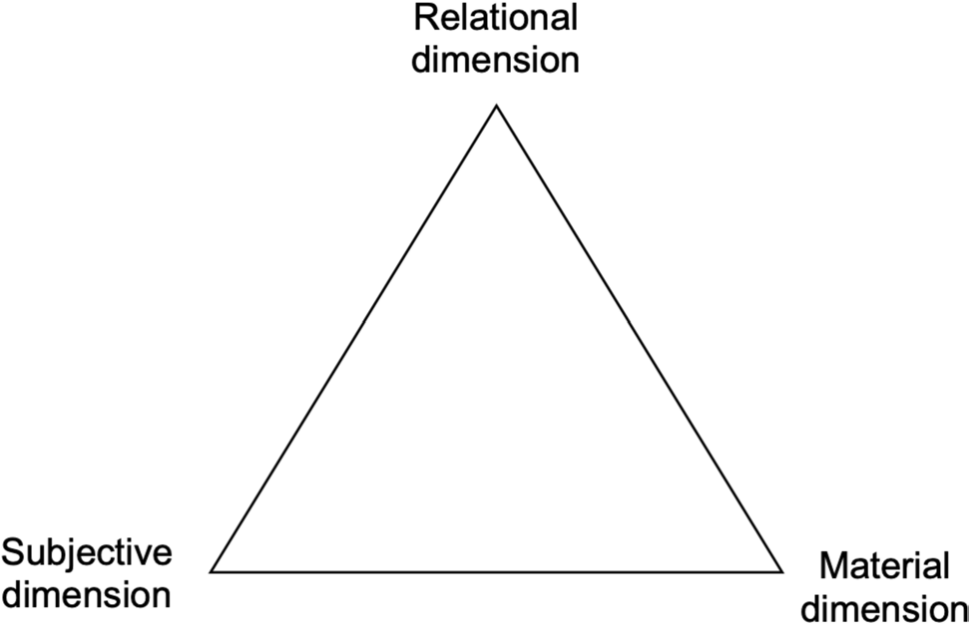
The three dimensions of the social wellbeing approach (adapted from White 2008)

The social wellbeing approach is represented by a triangle composed of material, subjective, and relational needs (Fig. 1). As illustrated in Table 1, the material dimension includes elements that fulfill physical needs; the subjective dimension encompasses psychological, emotional, and cognitive needs and wants; the relational dimension is concerned with the quality of connections with human and non-human others. The three dimensions of social wellbeing should be seen in heuristic terms, as key inter-related points of reference for understanding diverse perspectives on what gives meaning and satisfaction in life. As perceptions of wellbeing may vary fundamentally from one person to another, and between research participants and researchers (Gough and McGregor 2007; White 2008), and as wellbeing is deeply contextual and positional (Weeratunge et al. 2014; Johnson et al. 2018), a non-prescriptive approach to use of the dimensions of social wellbeing is appropriate. Social wellbeing analysis, in other words, recognizes the variability in material, subjective, and relational dimensions of wellbeing and their fundamentally interwoven character.

**Table 1:** Examples of the three dimensions of the social wellbeing approach (adapted from White 2008: 11)

Material	Subjective	Relational
Income, wealth, and assets	Values, norms, and morals	Social relations
Employment and livelihood activities	Understandings of the sacred and faith	Social and cultural identities
Education	Hopes and fears	Relations of love and care
Physical health and (dis)abilities	Aspirations	Networks of support
Access to services and amenities	Sense of belonging	Relations with the state: law, politics, and welfare
Environmental quality	Sense of meaning/meaninglessness	Conflict and violence
Provision and regulating ecosystem services	Levels of (dis)satisfaction	(In)security
	Trust	Collective action and agency
	Happiness	Power relations
		Social inequalities
		Relationship with the environment and with nature

In this paper, we are particularly interested in how identity, values, and social relations inform perceptions of wellbeing in a small-scale coastal Brazilian fishing community. Identity is a core constituent of different approaches that have influenced the emergence of the social wellbeing approach, including the economics of happiness and gender studies (Weeratunge et al. 2014). Identity links individual subjective aspiration with the relational dimension of collectively held symbols, values, and characteristic activities. Social relations are the mechanism by which identities are affirmed and challenged. In what follows, we present findings on the relationship between wellbeing and identity in two neighborhoods, Lázaro and Saco da Ribeira. We focus on two overlapping identities in particular: Caiçara and small-scale fisher. These identities are defined in relation to a set of activities and values that are central to subjective and relational wellbeing, and which frame a particular approach to material wellbeing. A short way to encapsulate elements of Caiçara small-scale fisher identity is their own term *vida simples* (living a simple, modest and humble life). As we elaborate in the “Commonalities and contradictions in expressions of wellbeing among small-scale fishers in Ubatuba” section below, however, neither vida simples nor the two facets of identity to which we link it should be seen as monolithic. Our research reveals a range of cross-cutting tensions and contradictions among the Caiçaras of Ubatuba that reflect social and economic differences and exposure to ongoing development and change in the region. Social wellbeing provides a methodology that can effectively address these ambiguities and variations in identity.

## Study area and people

**Fig. 2:**
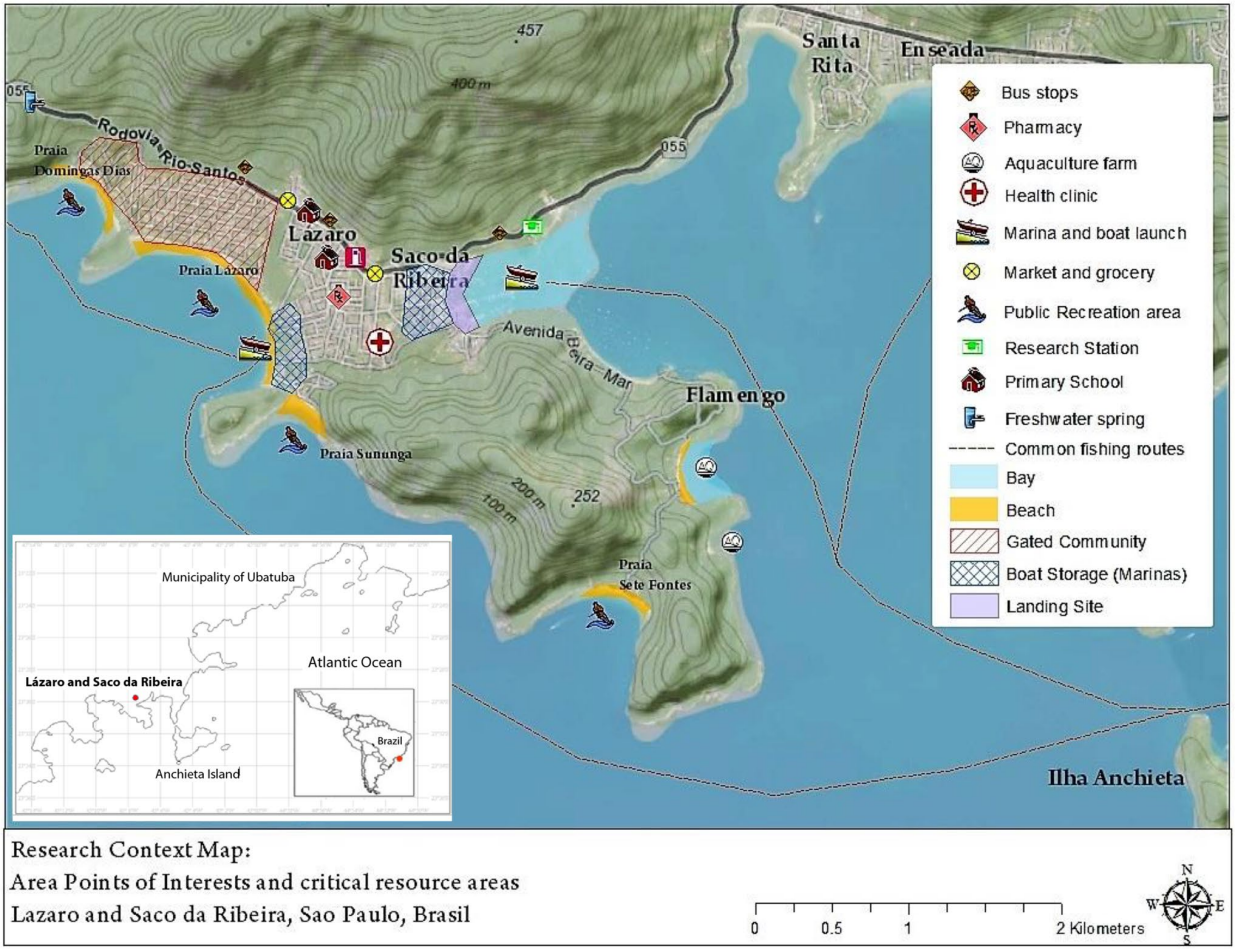
Research context map of Lázaro and Saco da Ribeira (Municipality of Ubatuba) with sites of interest and main fishing routes. Map created by the first author, using ESRI Geographical Information Products World Imagery (based on sources: ESRI, DigitalGlobe, GeoEye, i-cubed, USDA FSA, USGS, AEX, Getmapping, Aerogrid, IGN, IGP, swisstopo, and the GIS User Community)

The study area is situated in southeastern Brazil, on the north coast of the state of São Paulo, within the municipality of Ubatuba. This research took place in a community composed of two neighborhoods: Lázaro and Saco da Ribeira, located on the southern coast of Ubatuba (Fig. 2). The community is composed of a mixture of local and outsider residents, as well as seasonal tourists. At the time of the research, over 500 households resided in the two neighborhoods, according to data provided by the local clinic. This research focuses on the fishing community living within the two neighborhoods, which was composed of 44 households.

Over two thirds of small-scale fishers in the area used diverse fishing gears, depending on seasonality and commercial species availability. These included small gillnets (surface, mid-water, and bottom-set nets), hook and line, hand jigs, and small trawlers. Other than trawler fishers, most fishers owned their boats and gears. Out of 15 trawling fishers, five (33%) did not own trawlers and instead worked as a hired captain or as a crew member for boat owners. Several households had more than one type of boat: canoes, skiffs, and small trawlers, and it was common for fishers to shift boats depending on the resource targeted (Leite 2018). While the study focused on the households which relied primarily on fishing for their livelihoods, only a third of the fishers were fully dedicated to fishing activities (70% of these being trawler fishers). The other two thirds fished part-time and considered fishing as one part of a mixed livelihood portfolio. The great majority of fishers consumed part of their catches and shared it with family and friends. Some had retirement pensions, and some rented out accommodation seasonally to tourists.

The Lázaro and Saco da Ribeira neighborhoods host one of the largest fishing landing sites of the municipality, where fishers from many other states in the country also land their catches. Catches landed at the site include white-mouth croaker (*Micropogonias furnieri*), sea-bob shrimp (*Xiphopenaeus kroyeri*), sardines (*Sardinella spp*), pink-shrimp (*Farfantepenaeus brasiliensis* and *F. paulensis*), and blue runner (*Caranx crysos*), among many others. The bay also hosts several marinas and boathouses (Leite 2018). The study area is serviced with supermarkets, drugstores, small local shops, and public transportation. The community also has a public daycare and a school (primary and secondary) and a local public clinic offering basic health care. Many guest houses, run by outsiders, compete with locals offering rooms and houses to rent.

The Caiçaras, who inhabit the southeastern Brazilian coast, originate from a mix of Indigenous inhabitants, Portuguese colonists, African former slaves, and other immigrants (Begossi 1998). Traditional local livelihoods included shifting cultivation, fishing, hunting, and extraction of forest products (Diegues 2004; Denadai, Gonçalves, and Turra 2009). During the 1970s, the construction of the Rio-Santos highway brought an influx of immigrants and tourists to Ubatuba. Consequently, tourism-related livelihoods gained great economic importance for locals (Hanazaki et al. 2013). Simultaneously, accelerated land speculation for beachfront properties led to significant conflicts over land, resulting in many Caiçara families selling their properties and moving to suburbs along the highway. This rapid shift conveyed a spectrum of changes for Caiçaras, including losses and sales of land; immigration and emigration; religious changes driven by the arrival of many evangelical churches in the region; and conservation initiatives including the creation of parks, marine protected areas and non-fishing areas, closed seasons for target species, and gear restrictions, all of which had important impacts on the fishing communities’ wellbeing.

All protected areas were established as top-down arrangements with no prior public consultation. In Ubatuba Municipality, over 80% of the land area was allocated to the Serra do Mar State Park in 1977, restricting fishers from using their other livelihood sources such as hunting and shifting cultivation in the forest. In the same year, Anchieta Island and its surrounding waters — a highly favored and accessible fishing area for Lázaro and Saco da Ribeira fishers — was declared a State Park (i.e., no take protected area). Moreover, a sustainable-use marine protected area (Área de Proteção Ambiental do Litoral Norte de São Paulo), with 236,047 ha, was established in 2008 encompassing almost all fishing spots used by Lázaro and Saco da Ribeira fishers. These top-down measures led to much conflict between fishers and authorities (Leite 2018). Although the three protected areas do have consultative management councils where fishers may have a seat and some conflicts are negotiated, the ultimate decision rests with the state government agencies and conflict resolution takes a lot of time out of fishing.

## Methodology

This paper derives from a larger PhD project examining wellbeing (this manuscript), multi-level resilience (Leite et al. 2019), and the relationships between them (Leite 2018). The PhD study was part of an international collaborative project focused on community-based resource management and food security among Caiçara fishing communities in coastal Brazil (e.g., Begossi and Lopes 2014; Hanazaki et al. 2013).

The study reported here adopted a mixed methods approach embedded within an overarching ethnographic approach (see details below). Within this, the social wellbeing approach was followed as a guiding body of theory, but the specific methodology was adapted from Coulthard et al.’s (2015) handbook *Exploring wellbeing in fishing communities* rather than following the handbook exactly. This enabled us to take advantage of an opportunity for longitudinal data presented by the larger project’s survey data and allow the set of methods and specific procedures and questions asked to evolve sequentially so that findings from each method informed the others. Some of the methods were directed to gathering overall wellbeing perceptions (e.g., participant observation, open-ended interview about life history), while others focused more specifically on material, subjective, or relational dimensions of living well (e.g., household surveys, semi-structured interviews). While many of the methods were adapted from Coulthard et al. (2015), the household survey was adapted from Hanazaki et al. (2013) which investigated livelihoods, wellbeing, and food security among Caiçaras in Southeast Brazil. One method, focus groups, took a more significant role in our research than it has in Coulthard et al. (2015), following recognition of a gap in information about community dimensions. Thus, the social wellbeing approach was not applied mechanically, but used flexibly to understand the context-specific ways in which people perceive wellbeing.

Fieldwork was carried out from June 2014 to July 2015. A validation visit was conducted in May 2016.

Among the 44 households fishing households in the community at the time of research, 41 volunteered to participate in this research, i.e., the survey and other methods. Of the three households that did not participate, one was due to unwillingness of the household head to participate in the research, one due to sickness of a household member during the research period, and the third due to difficulties in finding a time when the participant was available.

Here, we are considering fishers as those individuals who actively fish on a regular basis as a source of income. Recreational and occasional fishers were, therefore, not included. The sections below explore the five data collection methods used, providing detailed information on the type of data collected, followed by a section on data analysis. Table 2 summarizes the methods and numbers of participants, in their sequence. The results of each method informed refinement of the subsequent methods.

**Table 2:** Summary of methods used

Methods (in sequence)	Purpose	Sample type and number of participants
Participant observation	Rapport building, learning by doing, and learning from observation	Community-wide in multiple spaces; participation in boat and fishing trips, community events, informal conversations, and day-to-day interactions, as well as observation in English classes offered free of charge to community members
Household survey	Build community profile	41 out of 44 fishing households in the community (participation rate 93%) One adult interviewed in each household, either the fisher or the fisher’s spouse
Open-ended interviews including narratives and life histories	Understand fishing as a way of life, values, worldviews, and life-changing events	Purposive sample (10 older men and 6 older women, total 16)
Semi-structured interviews including Global Personal Generated Index of Quality of Life (GPGI) and Relational Landscape methods, adapted from Coulthard et al. (2015)	Gain deeper understanding of values, identities, social relations, gender issues, and institutional engagement	Purposive sample of couples, with husband and wife interviewed separately, and of some single fishers (18 older men (12 married and 6 single) and 12 older women, total 30)
Focus groups	Address information gaps in community level processes and engagement in fisheries governance; relational wellbeing	Three groups. Purposive samples (11 older men; 6 older women; 7 younger people (4 male, 3 female), total 24)

### Participant observation: getting familiar with the fishing community and its members

**Fig. 3:**
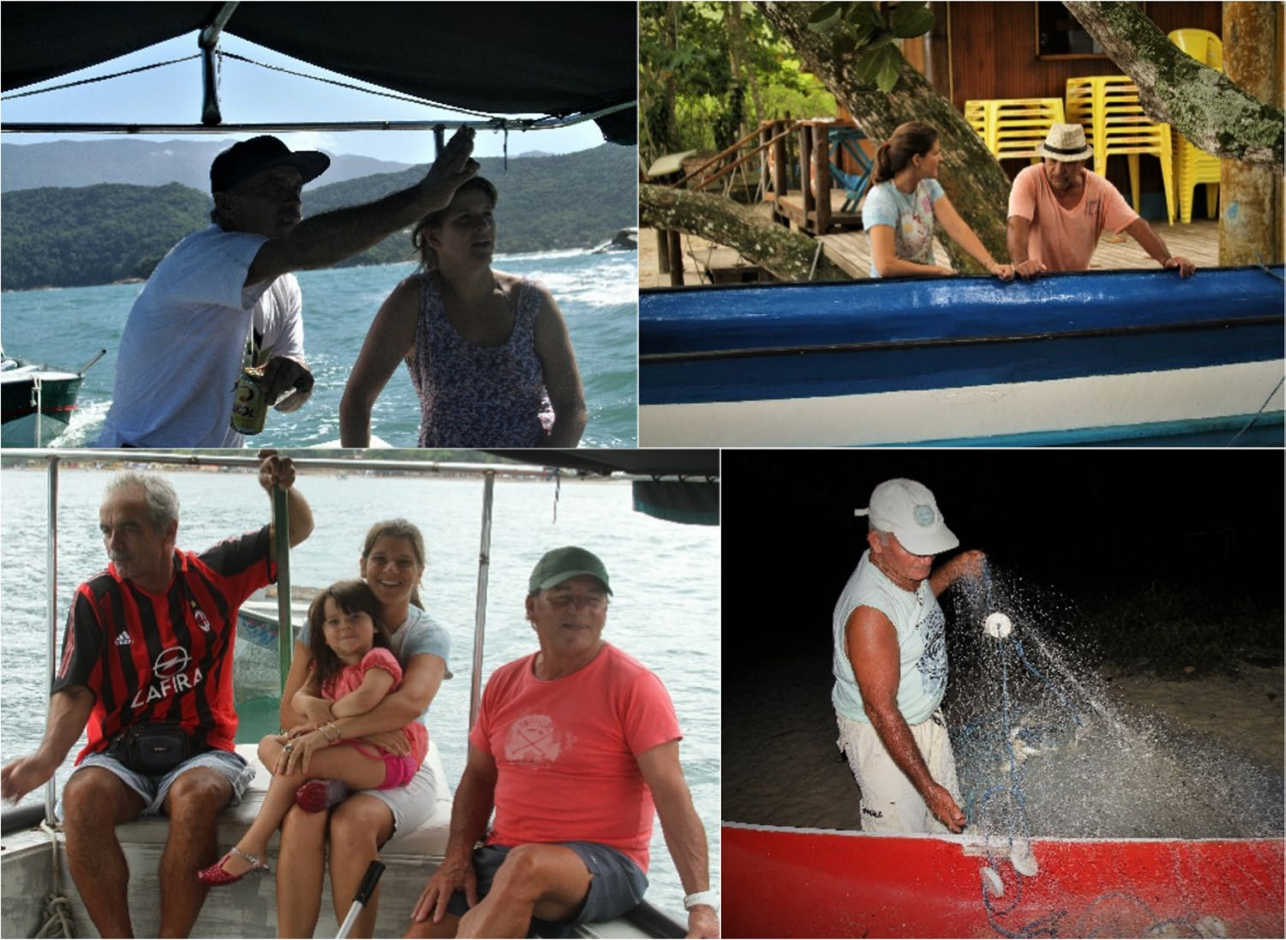
Interactions during participant observation. Photos: Marta Leite and Connor Jandreau

Wellbeing research requires the researcher to build strong rapport with participants in order to access the richness of their values and ways of life. With this principle in mind, the first author and her family resided in the community, renting a house from a respected local fisher. The fieldwork was initiated with an exclusively participant observation phase, aimed at gaining familiarity with the community members and, ultimately, the research participants. In participant observation, the researcher interacts with the people being studied in their daily lives (Creswell 2009; Hay 2008). This interaction allows the researcher to better understand their worldviews (Yin 1994) and cultural contexts. Moreover, it helps participants to feel more comfortable with the researcher’s presence, and consequently reduces the problem of reactivity (participants changing behaviors due to the awareness of being observed) (Bernard 2006). A significant amount of time was dedicated to participation in boat and fishing trips, community events, informal conversations, and day-to-day interactions, as well as English classes offered free of charge to community members (Fig. 3). Participant observation was continued throughout the entire period of fieldwork, alongside subsequent methods, and included the entire population of the community.

### Household survey: contextual and background information on participating households

Household surveys can be used to produce statistics about a target population (Fowler 2014; Hay 2008). They may also be used — with or without statistics — to produce contextual, or background information about a set of people (Fowler 2014), as was the case in this study. We interviewed one male or female adult per participating household (thus 41 people, reporting on their households).

We chose the questionnaire of Hanazaki et al. (2013) rather than that of Coulthard et al. (2015) because it permitted comparison with other data collected within the overarching project, while addressing our needs for background data on the participants and initial wellbeing information that could be enriched through the later methods. The household surveys were essential to collecting contextual information such as household composition, household members’ ages, education level, gender, occupations, and livelihood portfolios. We also gathered data on fishing gear and fishers’ work history (e.g., number of years spent fishing, degree of dedication to fishing activities, local knowledge, boat, gear and boat ownership, main target resources and fisheries contribution for the household income) and data on food security (e.g., fish and catch consumption, food sharing within the community, vegetable and fruit production, existence of food insecurity within households). Finally, data regarding financial wellbeing and basic needs were also collected during the household surveys.

The household survey identified 48 fishers, aged 18 to 89 years old. Of the 48 fishers, 38 were adult men, five were women, and five were male youths aged between 18 and 28 years old. The five fisherwomen fished together with their husbands and also contributed to pre- and post-harvest activities (these were all from households with just two or three members, i.e., these women had no children or one child at home). In addition, eleven women, who did not fish, were involved in fishing related activities, such as pre- and post-harvest activities and household budget management.

### Open-ended interviews: narratives and life histories

Open-ended interviews were used to explore *narratives* of specific challenging life-events, and *life histories*, to connect the lives and stories of individuals to their values and worldviews, allowing for a deeper understanding of the local fishing way of life. Life history can be considered a type of narrative, or an autobiographical narrative, yet not all narratives are life histories as they can relate to a specific event (Hatch and Wisniewski 1995: 133). Lejano et al. (2013: 63) suggest “it is the stories people tell that connect them and others, including non-human parts of the environment, into a coherent whole, and provide meaning.”

We purposively selected 16 participants (ten men and six women) to ensure representativeness of all participant groups (e.g., different age groups, gender, type of fishery, social strata, and household composition). We asked the question “Could you please tell me about your life as a fisher, or as wife of a fisher, and the challenges and opportunities you faced?”

### Semi-structured interviews: a deeper understanding of values, identities, and social relations

Based on data collected under the previous methods, semi-structured interviews (Creswell 2009; Denzin and Lincoln 2000; Hay 2008) were conducted to explore participants’ understandings of wellbeing. While the open-ended interviews gave participants room to direct the interview to topics of their interests, the semi-structured interviews favored the interests of the researchers, allowing for questions to be targeted in a more formal manner. Still, the semi-structured interviews maintained a degree of flexibility to explore other related topics that emerged during the interview.

The interviews were conducted with both fishermen and fisherwomen and their spouses (if the case) separately, totaling 30 participants, 12 women and 18 men. The selection criteria called for the majority (over 60%) of the fishing community households to be interviewed, while ensuring also that these interviewees spanned different age-groups, gender, type of fishery, education level, and social status found in the fishing community. All interviewees had been involved in the previous stages of the research (household survey and/or open-ended interview).

The interview questions were dedicated to deeply understanding the meaning of living well for different participants, including material, subjective, and relational dimensions of wellbeing. The Global Person Generated Index (GPGI) and the relational landscape methods were applied as part of the interviews, both adapted from Coulthard et al. (2015). The GPGI is designed to assess individualized quality of life measures for a holistic understanding of subjective wellbeing in developing countries (Martin et al. 2010). In the Coulthard et al. (2015) version of this method, participants are requested to first list five of their most important areas in regard to wellbeing; then to rate their level of satisfaction with each of these areas; and last to expand points they consider relevant regarding their self-selected areas. Levels of satisfaction were represented with numbers from 1 to 5, 1 being “bad,” 2 “poor,” 3 “OK,” 4 “good,” and 5 “excellent.” Here, it is important to highlight that only participants who select a specific area of wellbeing get to rate their satisfaction levels within that area. For example, if a participant chooses “Money” as a wellbeing area, then that participant gets to rate their level of satisfaction with “money.” We adapted the GPGI method in two ways. First, participants were asked to rank the five most important wellbeing areas they had selected, in order of importance. This process often involved the reordering of wellbeing areas as participants contemplated and evaluated important wellbeing markers. The researcher gave time for such reflections. Second, participants were requested to elaborate in greater depth the areas where the levels of satisfaction were scored low through probing as to “what would be needed for this area to receive a better score?” Therefore, the researcher used the GPGI as a prompt to elicit further reflection from participants with interest in particular on areas of relatively low satisfaction.

The relational landscape is our revised name for what Coulthard et al. (2015) call the Governance Relationship Assessment method. We have modified the name to reduce the emphasis on how social relationships “govern” social life. We are more interested in the instrument as an open-ended way for research participants to subjectively evaluate the quality of relationships important to them. We asked research participants to identify their most important relationships for the participants at several levels: household, extended family, fishing community, broader community, and formal institutions (including management agencies). The relational landscape can be depicted, as we have done in Fig. 8, using nested circles representing each level (from individual to governmental level). Based on answers to questions about satisfaction with each kind of relationship deemed important, we placed that relationship on the corresponding level with proximity to the top of the circle indicating relative positive satisfaction with the relationship. The innermost circle represents the research participant. The next circle (from inside moving out) refers to their relationship with other household members, the third circle represents their relationships with extended family members, followed by their relationships with the fishing community, the broader community and finally, the outermost circle refers to relationships with governmental institutions (for more details, see Coulthard et al. 2015).

### Focus groups: relationships at multiple levels

Lastly, focus groups took a more prominent role in our research than suggested by Coulthard et al. (2015). We used focus groups to help fill a data gap regarding community level processes and engagement in fisheries governance that surfaced over the course of the fieldwork. In particular, focus groups explored the relational dimension of social wellbeing. Two colleagues from a Brazilian university, both trained in facilitating focus groups, moderated the groups, with the first author present as recorder and observer.

All participants from the 41 households involved in the research were invited to participate. Three focus groups were conducted, with adult men (11 participants), adult women (six participants), and youth (seven participants, four male and three female) respectively. The meetings lasted an average of 2 h each. Focus groups were held in the community, in the house rented by the first author. Questions explored community-wide relationships and conflicts, group agency, and fishers’ participation (or lack thereof) in fisheries governance (see Table 2).

**Fig. 4:**
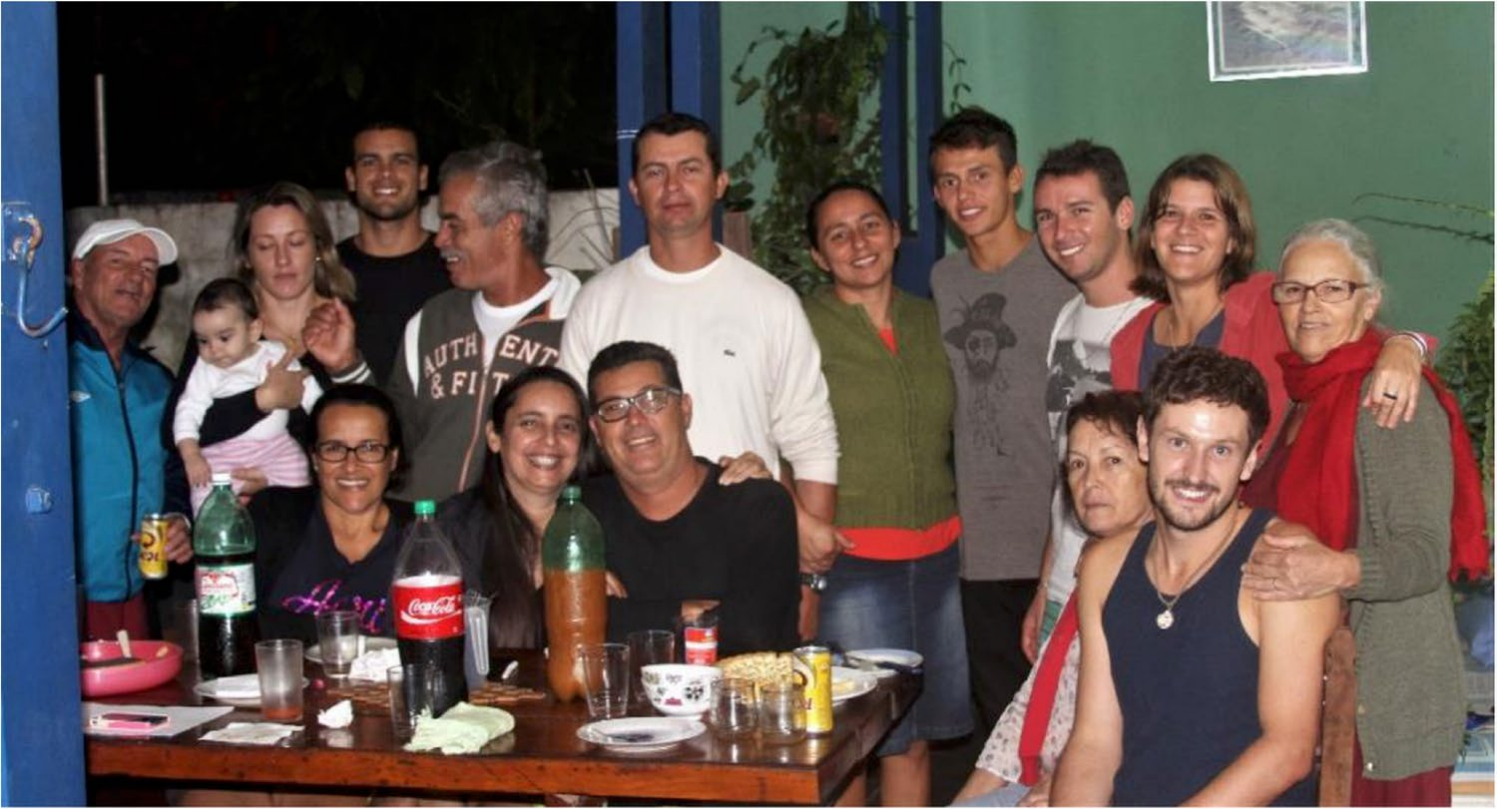
Gathering with participants prior to the researcher’s departure from the field site. Photo: Marta Leite

Near the end of the study, a community gathering was held with fishers and their family members (Fig. 4). The purpose of this gathering was to thank participants for their contributions to the research, as well as to offer a farewell party prior to the first author’s departure from the field site.

### Data analysis

The results were analyzed in two stages. Preliminary analysis in the field allowed emerging results to inform refinement of the subsequent methods. Upon return from the field, all results were analyzed deeply, method by method and across all methods, to synthesize findings relating to wellbeing, identity, and values, following the social wellbeing approach.

The household survey data was organized first in an Excel spreadsheet and descriptive statistics analysis was carried out for quantitative data. The survey qualitative data, as well as data from participant observation, open-ended interviews, semi-structured interviews, and focus groups, were all transcribed in Word documents. NVivo software was used to manage, organize, and analyze the large volume of data gathered across the five chosen research methods. In NVivo, the data was divided by source (data collection method), and then coded by topics (nodes), allowing for the creation of subcategories. The topics, and therefore codes, were predefined according to the three dimensions of the social wellbeing approach (relational, subjective, and material). Qualitative data were analyzed through triangulation of information coming from different sources and/or methods. Some counts of frequency of mention of specific themes and rankings of priorities in the semi-structured interviews were also made, to suggest relative importance accorded by the participants. Priorities included being healthy, money, God and faith, freedom to fish, friends and neighbors, family, sons and daughters doing well, relationship with spouse, land and house ownership, and job satisfaction. Since the semi-structured interviews are based on a purposive sample, of half of the adult participants in the study, it is not appropriate to generalize these counts to the entire population.

### Limitations

The scope of this in-depth study was necessarily confined to the time and cash budget of a doctoral study. The depth achieved was possible through previous studies, including those conducted as part of the IDRC/SSHRC funded International Chairs Initiative project, and good relationships in this community. As with all single case studies, points within the findings may or may not be generalizable to other places, in Brazil or elsewhere.

## Commonalities and contradictions in expressions of wellbeing among small-scale fishers in Ubatuba

The methodological diversity that we adopted in our research provided evidence to tell a distinctive wellbeing story about the Caiçaras of Ubatuba. Semi-structured interviews and participant observation suggest strong identification as Caiçara and small-scale fishers in ways that are closely aligned with key elements of the “vida simples” ideal. Moreover, results from the household survey suggest that these areas of identity provide important points of reference for how people justify their self-assessment of relatively high levels of wellbeing. The story is complicated, however, by more fine-grained subjective and social relational evidence derived through the GPGI component of the semi-structured interviews, participant observation, and the relational landscape method. It was clear that a positive association with a Caiçara small-scale fishing identity at the population level was subject to complex relational variations linked to gender, gears used, socio-economic position, and personal histories and experience. We present our findings with regard to Caiçara fisher identity in a layered approach that brings out evidence from different methods, beginning with broader findings about identity from the semi-structured interviews.

### Caiçara fisher identity

Our research formally addressed Caiçara and fisher identity through questions that we asked during the semi-structured interviews. Most importantly, we asked, “What does it mean to you to be a Caiçara?” and “What does it mean to you to be a fisher or the partner of a fisher?” Table 3 summarizes the descriptors participants used in the semi-structured interviews, and presents quotes illustrating participants’ self-identifications. We recognize that the answers to these questions are often idealizations and simplifications. They need to be contextualized in relation to historical and social conditions and to positionality. Nonetheless, the idea of what is a Caiçara and what is a fisher are important reference points in identity construction. Our argument and work in this paper turn on this effort to balance the influence of the Caiçara and fisher components of identity with the larger complexity of making a life in Lázaro and Saco da Ribeira.

**Table 3:** Identity descriptors used by Caiçara research participants

Caiçara self-identification	
Descriptor	Quotes
The people born on the coast	“Everyone born on the coast is a Caiçara, but many Caiçaras live like people from the city now.” “My daughter is the fifth generation born here. If she marries someone from outside, and they leave our place [the community], she will be different. Still, she will be a Caiçara, but not living a Caiçara lifestyle, you know?”
Modest and humble people Embrace a simple life	“We are simple; we enjoy the simple things of life.” “Caiçaras are humble.” “I would not exchange this life that I have for all the money in the world. People from São Paulo wanted to buy my house. They offered me a ton of money. I said: No thanks! This is my place, and here I will stay!”
Laid back and relaxed	“I prefer to work less and live my life in an easy way. Money makes people stressed, have you noticed?”
Welcoming of outsiders	“We welcome tourists; we depend upon them. If they do not show up, we struggle to pay our bills. So, we must welcome them always.”
Suspicious of strangers	“We Caiçaras take some time to trust people and bring them into our lives.” “Once you gain the friendship of a Caiçara family, you have good friends, but it might never happen too [laughs].”
Caiçara fisher identity	
Live in the present	“Future, for me, is the bills at the end of the month.” “Fishers have to be like that [live in the present moment], we never know if we will have fish in the net, so we have to live without worrying too much.”
Spiritual and/or religious	“Faith! We need to have faith that a good catch will come soon. If you do not trust that a good catch is coming, you can’t be a fisher because you lose hope.”
Freedom	“At sea, I am my own boss, on land there is my wife [laughs].” “Fishing is a source of money and pleasure. I do what I love, what makes me happy, and I decide when and how.”
Sense of humor	“We [fishers] like to make fun of everything! You must know that by now [laughs]. We make fun of you, as much as we make fun of our wives, kids, friends. We make fun of ourselves too!” “José magnifico [Magnificent Joseph] was the first one [fisher] to have a boat here. They say that one day he went fishing grouper and got the hook stuck in his finger. In the hospital, he said to the doctor "cut my finger off, but do not break my hook! [Lots of laughs]”
Holders of local/fishing knowledge	“A true fisher is the one that knows the sea, the wind, the weather and how and when to kill fish.”
Adventurous/courageous	“When you go to sea, you are never sure of how it will go. It is a new adventure every day.”
Apolitical	“Fishers are the most relaxed and disunited people that exist. Others fight for their rights, but us fishers, we stay quiet.”

Table 4 lists quotes collected during participant observation collected during informal conversations with non-Caiçara community members, conducted as part of the participant observation method.

**Table 4:** Descriptors of Caiçara fishers used by non-Caiçara research participants

Caiçara fishers as seen by non-Caiçaras living in the community	
Friendly	“They are normally nice and polite with us.”
Without ambition	“They have no ambition. They do not care for more comfort, not even for their kids. I am different.”
Uneducated	“They lack formal education; they are just like hicks. They have tradition, and culture, but not much education.”
Too peaceful	“They are not fighting for their rights. They are too laid back, too peaceful. They will go on just complaining forever.”
Naive people	“Caiçaras lost a lot of land in the past because they are too naive. People from outside took advantage of them.”

Our data indicates that Caiçara fishers and their families make reference to shared ideas of what constitute Caiçara fishing identity and way of life. Indeed, we found these reference points central to the conceptualization of wellbeing in Lázaro and Saco de Ribeira. Being laid back, the Caiçaras’ distinctive sense of humor, living in the present and therefore not worrying too much about the future, a strong faith in God and in the divine, the embrace of a simple and humble lifestyle, were not only cited by participants as self-descriptors, but were manifest in behaviors that the first author observed in day-to-day interactions with research participants during her fieldwork.

### Caiçara fisher identity in the broader fabric of wellbeing: reading between the lines of the Global Person Generated Index and relational landscape methods

The GPGI and relational landscape methods are aimed at eliciting individual assessments of the most important features of the lives of research participants that contribute to their subjective and relational wellbeing. Elaborations of how and why different features of life contribute to wellbeing (or illbeing) are not exclusively subjective or relational, however. Subjective, relational, and material aspects of wellbeing are interrelated. Reflections on relational dimensions of subjective experience, or the subjective or material implications of relationships, provide valuable insights into the diversity and complexity of identity. The GPGI and relational landscape results, in other words, contextualize and situate Caiçara fisher identities. Our application of these methods foregrounds the cross-cutting effect of gender identity in particular, but also brings out family and religion as complicating the subjective and relational aspects of Caiçara fisher identity. That identity is neither homogeneous nor complete unto itself.

#### Contrasting perceptions of life satisfaction by gender identity

**Fig. 5:**
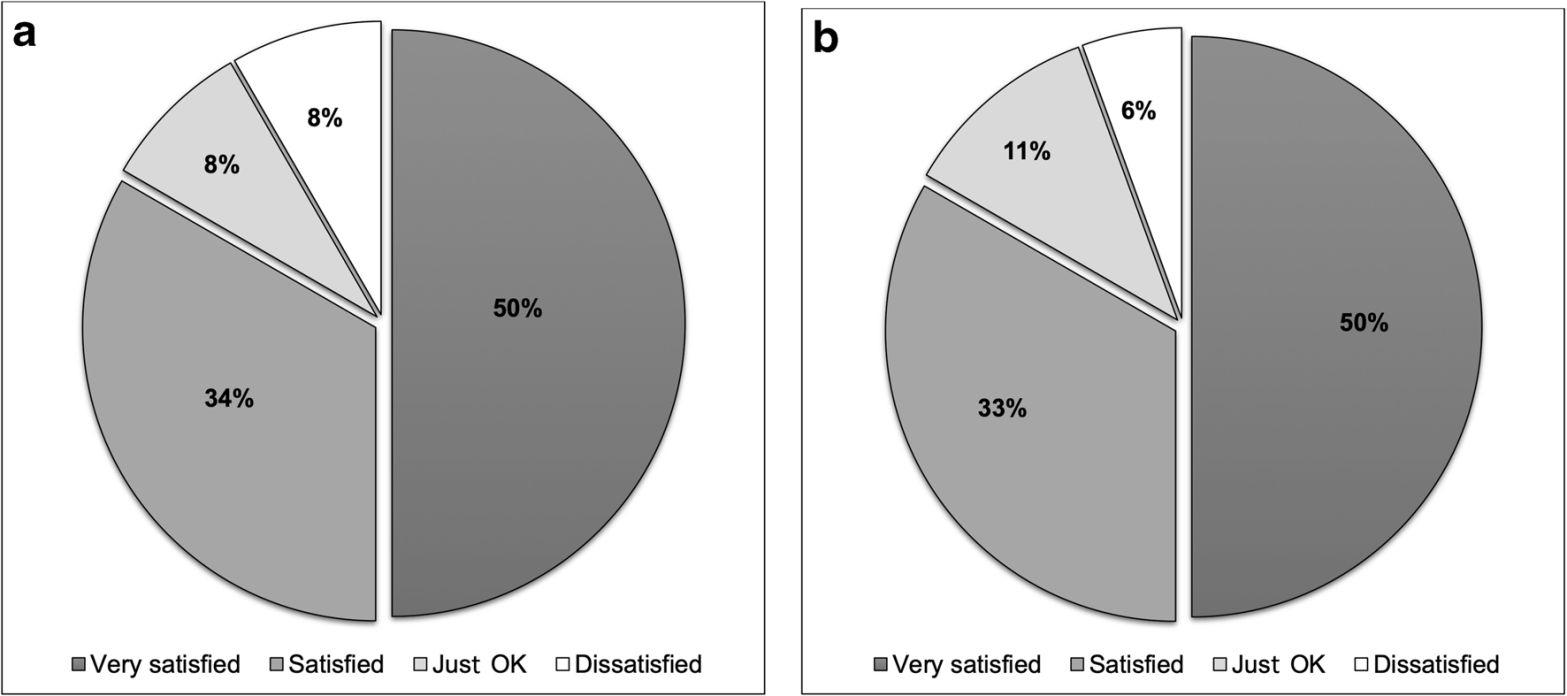
**a** and **b** Levels of life satisfaction by gender, *n*= 30 (18 male, 12 female) as given by research participants in semi-structured interviews

**Fig. 6:**
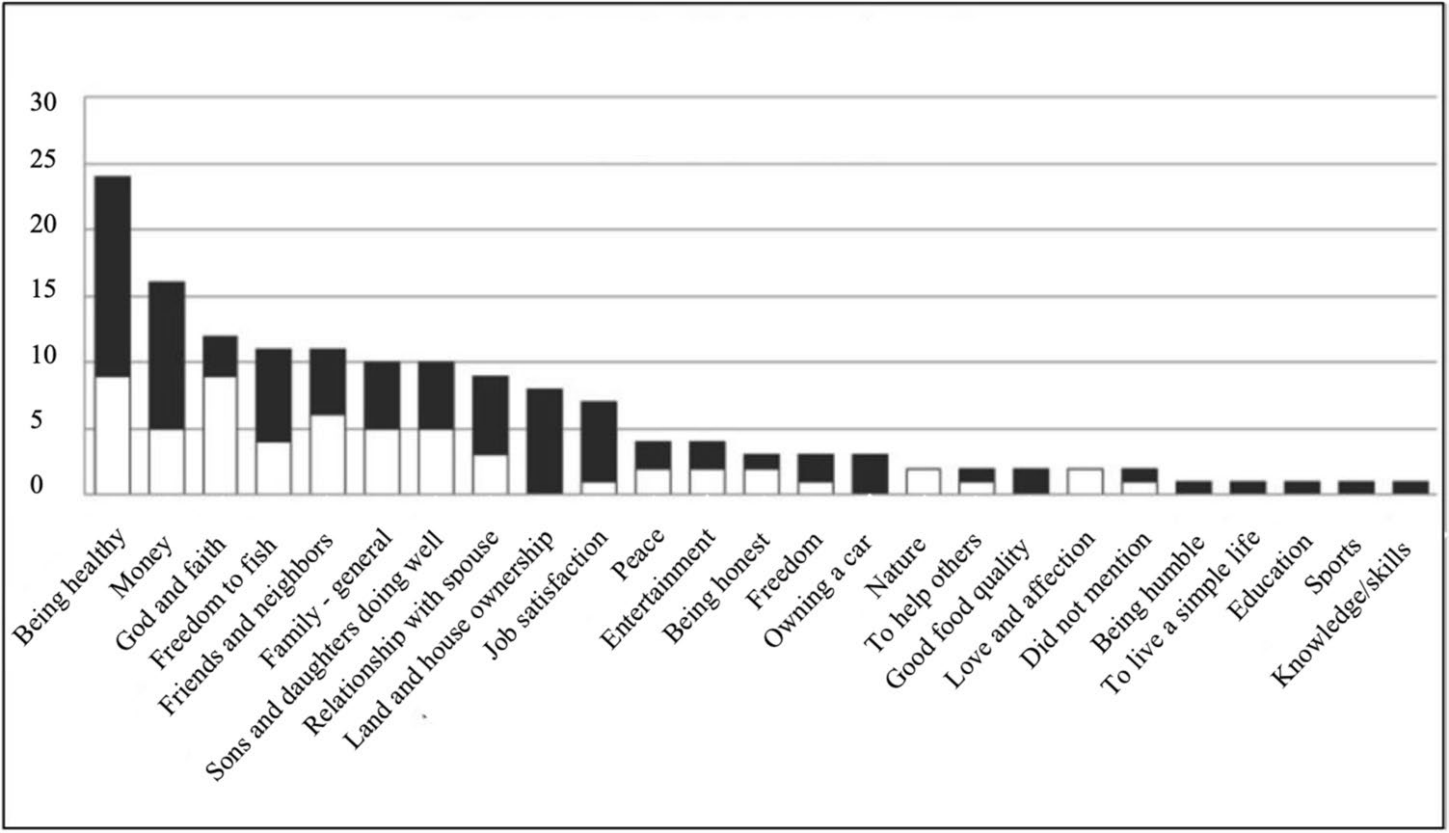
Important wellbeing areas by gender. Black represents males (*n*=18) and white represents females (*n*=12), total *n*=30

Significant distinctions surfaced when comparing levels and areas of life satisfaction by gender, as presented in Figs. 5a, b, and 6. In this paper, we will focus on areas that had more than 30% of mentions, by either men or women, or both. An analysis by gender revealed that for men, the prioritized areas were “being healthy,” “money,” “land and home ownership,” “freedom to fish,” “relationship with spouse,” and “job satisfaction.” For women, on the other hand, the prioritized areas were “God and faith,” “being healthy,” “friends and neighbors,” “family (in general),” “sons and daughters doing well,” and “money.”

**Fig. 7:**
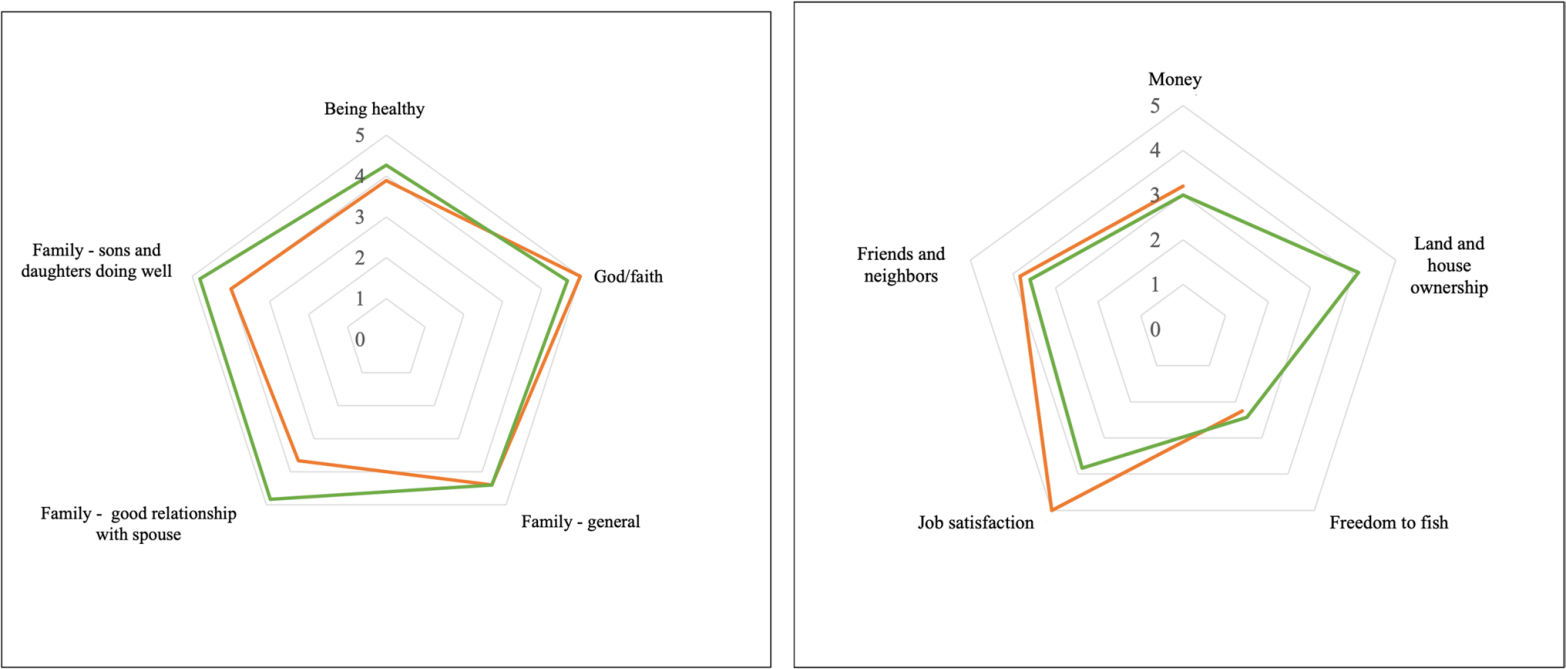
**a** and **b** The 10 most cited areas described by fishers and their spouses as important for wellbeing and corresponding levels of satisfaction by gender (*n*=30). In the figure, 1 is “bad,” 2 “poor,” 3 “OK,” 4 “good,” and 5 is “excellent.” Green represents men and orange represents women

Figure 7a and b present an overview of the ten main areas cited as important for participants’ wellbeing and the average levels of satisfaction cited during the GPGI method. The data are presented by gender. The ten wellbeing areas cited were divided into the two diagrams below to allow for better visualization. It is important to mention that the area “land and house ownership” was only cited by men, and the area “job satisfaction” mostly by men (only cited by one woman). The sub-sections below explore the ten most cited wellbeing areas in detail while grouping “family (in general),” “sons and daughters doing well,” and “relationship with spouse” under the overarching “family” area.

##### Health

The category “being healthy” was identified as the most important area for participants’ wellbeing (cited by 80% of participants). Interesting, a gender analysis demonstrated that men tended to value this category more than women (Fig. 6). Men also gave “being healthy” a larger weight, as 56% of men considered it the most important component for their wellbeing, while for women, the “being healthy” area was as important as the “God and faith” area. An explanation given by a male participant for this gender difference was that fishing demands physical effort, and therefore, “being healthy” is essential for fishers’ day-to-day routines. Another central point is that for many participants, “being healthy” meant more than just physical health, also incorporating components of mental health, including psychological and emotional features. Indeed, fishing was pointed out as central to fishers’ mental health and subjective wellbeing. This is explicit in a quote from a male adult fisher: “The waves wash our problems away.” Many fishers mentioned during the semi-structured interviews that fishing and the pleasure of being at sea represented a source of stress reduction and relief from day-to-day struggles.

##### Money

While the “money” area demonstrated general importance according to a number of people who listed it among their top five areas of wellbeing (see Fig. 6), in the ranking of the areas of wellbeing, only one male participant ranked it as the most important area for wellbeing and only 22% of men and 16% of women ranked it as their second most important area (Fig. 7a, b). This finding is interesting in terms of gender roles and social change. Even as women were increasingly engaged in paid jobs, men seemed to experience more pressure to provide financial security for the household. As a fisherman, and his wife, explained during individual interviews, respectively:

My wife works, but the responsibility for paying our bills is mine. I appreciate her help, but that is a responsibility of the men.

I work because fishing is not enough to pay the monthly bills. But my husband is too proud to admit it.

Other seemingly contradictory points that emerged from the “money” area analysis were that while many participants — during semi-structured interviews — declared money as important mainly as a means of meeting their basic needs, the levels of satisfaction within the category, identified by the GPGI method, were not necessarily high in spite of basic needs being met among most households interviewed. Therefore, while general levels of life satisfaction were high (for both men and women) as demonstrated earlier in Fig. 7, more targeted questions about “money” gave a nuanced, if not contradictory perspective.

The analyses of levels of satisfaction identified through the GPGI method demonstrated that while placing greater value on “money,” men also tended to be less satisfied than women. Men declared their levels of satisfaction with “money” primarily as “OK,” and to a lesser extent “poor,” while women indicated “good” and “OK,” and only 20% of women declared it as “poor.” Here, it is important to emphasize that only participants who declared “money” as one of the five areas with the most relevance for their wellbeing were then asked to rate their levels of satisfaction with it. Therefore, almost half of participants, those who did not declare “Money” as one of the five most important areas, are not represented in the satisfaction rating analysis for this area, suggesting that many participants are content with a modest income and their way of life. In further exploring those individuals who declared their “money” satisfaction levels as “OK,” most were fishers who did not have alternative sources of income. In other words, lower levels of satisfaction with “money” were common among individuals who had fishing as their sole source of income. Again, households that did not have extended families living in the community also tended to indicate the “money” area as important for their wellbeing, and present lower levels of satisfaction with it. Therefore, there is a strong indication that individuals experiencing tougher financial circumstances were the ones relying primarily on fishing for income or those with weaker networks of support within the community.

##### God and faith

While the two first themes in Fig. 7, “health” and “money” had higher representation among men; the category “God and faith” was most frequently cited by women. In fact, 75% of women interviewed prioritized the area as important for their wellbeing, compared with only 17% of men. Moreover, almost half of the women interviewed indicated “God and faith” as the most important area for their wellbeing as compared to a single male participant.

This data seems to suggest that men did not necessarily value “God and Faith” as an area of wellbeing. However, it is important to reaffirm that while an area may not be prioritized as one of the *five* most important areas for an individual’s wellbeing, such absence does not necessarily mean it is not valued by participants. The fact that the GPGI method only allowed for the recognition of five areas must be acknowledged as a limitation for the identification of other possible important areas that do not necessarily surface in the method. This is especially relevant for the “God and faith” wellbeing area, as the “relational landscape” method and several complementary questions made to participants during the semi-structured interviews suggested a strong relationship between Caiçara male fishers and faith (Fig. 6). Despite its higher significance for women, Caiçara people are, in general, very spiritual and/or religious. As a male fisher explained:

Women are more dedicated to the church, but men have a lot of faith in God too. Fishers need faith: faith in God, and that he [God] will help us bringing home a good catch. If a good catch does not happen today, we need to have faith it will tomorrow, you know?

Fishers referred to fishing also as a source of relief for day-to-day problems, and therefore a source of psychological resilience and subjective wellbeing. In addition to “being in and with nature” (male adult fisher), being physically active and meeting the constant challenges of the sea and the catch, important subjective and relational aspects of fishing activities reported were stress reduction, positive emotions, social integration, feelings of freedom, and pride in being a fisher. All of these points emphasize that the sea acts as a source of subjective wellbeing for Caiçara fishers. The importance of religion and spirituality for participants is also reflected by the general levels of satisfaction with the “God and faith” area, where all women who cited the area declared their levels of satisfaction to be “excellent,” and all men said it was either “excellent” or “good.” Caiçaras — like the large majority of Brazilians — were mainly Catholic until the 1970s, when evangelical churches started to establish themselves in many parts of the country including coastal communities. At the time of the fieldwork (2014–2015), there were four different churches in the community, one Catholic and three evangelical.

While churches were identified as central networks of support in times of illness, loss, death, and financial hardship, as well as supporting recovery from substance abuse, the diversity of Christian belief systems was also linked to diminished community cohesion. During the women’s focus group, participants highlighted that the growth of religious diversity has had at least three distinct impacts in the community: (1) it has shrunk networks of support, to within same-church communities; (2) it has contributed significantly to the subjective wellbeing of religious individuals, including as an important support system for substance abuse recovery, both for men and youth; and (3) it has weakened relationships at the broader community level as the growing divergence in religious belief systems has had a strong impact on relationships at the community, extended family, and even household levels:

Yeah, it [religion] divided [the community] a lot. I feel it in my own skin, with my family. We used to have many festivities together, now that half of the family is evangelical, we do not get together on New Year’s Eve and many other holidays. (Catholic woman, in the women’s focus group)

##### Freedom to fish

The area “freedom to fish” was expressed as one of the five most relevant wellbeing areas for over a third of participants (Figs. 6 and 7). Furthermore, 10% of participants declared “freedom” (in more general terms) to be essential for their wellbeing. Almost half (44%) of the men (eight) mentioned freedom to fish, while 33% of the women (four) also cited this area. Three of these women were fishers and one was the wife of a fisher. Indeed, our data suggests that “freedom to fish” is more than an area relevant to wellbeing; it represents a core value for the fishing community. Again, as observed through multiple methods, the fact that the area was not selected as one of the five most important wellbeing themes does not imply that many fishers do not feel the constraints imposed by fisheries management on their day-to-day freedoms. The high levels of dissatisfaction with the area illustrate this point (Fig. 7). “Freedom to fish” was the wellbeing area with lowest satisfaction levels (“OK,” and “poor”). Fishers’ frustrations with imposed limitations on their fishing activities, either by the establishment of no-take protected areas or gear restrictions (see also Leite et al. 2019), are reflected by numerous statements like the following one:

Nowadays we fish with fear. We can’t fish here; we can’t use that net… We can’t go fishing when, where and how we want anymore. They [enforcement agents] control everything we do. I fish because I have been a fisher my whole life. If not, I would probably give up, you understand? The [government agency responsible for fisheries management] wants to extinguish us; they do not care about artisanal fishers.

A way of dealing with the issue of imposed fishing restrictions commonly mentioned during interviews and informal conversations was making the decision to fish illegally, particularly in the surroundings of the Anchieta Island, a historical fishing area adjacent to the community, where fishing has been banned since 1970 due to establishment of a state park (no-take protected area):

If they [government agency responsible for fisheries management] do not let me fish, I will “steal the fish” [fish illegally]. Not because I want to be an outlaw or a criminal, but simply because I am a fisher, and fishers fish. That is what we are [fishers], that is what we do [fishing].

Nevertheless, there are severe consequences for fishers if caught fishing illegally, such as arrest and loss of fishing gear. Furthermore, such incidents result in the marginalization of small-scale local fishers more generally.

##### Relationships with neighbors and friends

Relations with neighbors and friends were central to women’s wellbeing. Fifty percent of female participants declared “relationships with neighbors and friends” as one of the five most important areas for their wellbeing (Fig 6). The same was declared by 28% of men. While participants commonly cited this wellbeing area, it rarely ranked higher than fourth in order of importance for men and women alike. Social life, while remaining an important part of wellbeing for most participants, is reflected in this data as trending downward with diminished community cohesion over recent decades. Still, relationships with friends and neighbors played important roles in the community in terms of kinship ties, including the sharing of fish, vegetables, and fruits, looking after others’ children, and fishing together. Friendship ties also were declared, mostly by women, as important for emotional support in hard times, especially by women who mentioned having concerns regarding their domestic lives that they did not feel comfortable sharing with family members.

For those participants who included “friends and neighbors” as one of the five most important areas, levels of satisfaction varied. A strong trend that surfaced from this data was higher levels of satisfaction held by members of extended families who had lived in the community for many generations (normally for three or more generations), and lower levels of satisfaction in fishing families that were the first generation in the community. Marriages between members of extended families living in the community were quite common and played a role in maintaining networks of support among the traditional local families. There were, at the time of the fieldwork, eight different extended families living in the community for more than three generations (in many cases for four or five). These families were mostly Caiçaras that still held land in the study area, and that passed land, boats, fishing knowledge, and skills down through the generations.

##### Family

If considering the three categories related to family: “family (in general),” “relationship with spouse,” and “sons and daughters doing well” as one broad “family” category, it then becomes the most cited area (Fig. 6) as it outnumbers both “being healthy” and “money” areas in total number of citations. Indeed, further analysis of numbers of individuals that considered some aspect of “family” as one of the five most important areas for their individual wellbeing results in a tally of more than 96% of participants.

The central role of the family unit, either represented as immediate family members or as extended family, is fundamental for Caiçara identity and culture, in addition to its importance for wellbeing. When approaching levels of satisfaction for the three categories related to family, some interesting findings surfaced. While both men and women demonstrated high levels of satisfaction with “family” as a general area, areas related to “sons and daughters doing well,” and “relationships with spouse,” showed that men tended to present higher levels of satisfaction in both areas than women (Fig. 7). Participants identified indicators of “doing well” for their young children and teenagers in activities such as attending school, earning good marks and not being involved with drugs, while for their young adult sons and daughters, participants’ indicators of “doing well” included being “well-married,” “having a job and decent income,” and “being good parents to their own children.”

An explanation, based on interviews with some of the participant women, might be that mothers tended to have higher expectations for their children than men. Perhaps one reason is the perception, often held by men, of less need for formal education, and rather an emphasis on local knowledge, required for fishing activities. Some women also demonstrated greater anxiety over their children’s futures, as exemplified by women’s greater tendency to save money, while men found themselves more often in “the present moment” and not as concerned with future expenses.

Finally, the men who demonstrated higher levels of satisfaction with the category “relationship with spouse” were, for the most part, according to the open-ended interviews, older fishers (60 years old or older) who have engaged in larger scale fisheries in the past (which required being away from family for several weeks), and/or wrestled with alcoholism in their younger years. For these men, being out of the family setting for long periods in their past lives as fishers, and having their wives and children patiently waiting for them back home, seemed to contribute to valuing their relationships with their wives nowadays:

This woman is strong, you know? She stuck by my side through hard times. She forgave my absence, my drinking, and rudeness in the past, and I respect her for that! (Older male fisher)

Nevertheless, elderly women struggled during these times; they shared memories of being insecure about when their husbands would be back home, their safety at the sea, if the income would be enough to fulfill their family needs, and furthermore having always to readapt to their presence and absence in the community:

We were always waiting for him to come back, but I will admit to you, sometimes I couldn’t wait for him to leave for the sea again. He came home and thought he deserved to rest, he did not realize how much work it was for me to stay on land with our seven children.

##### Land and house ownership

Land ownership is a very important aspect of wellbeing among Caiçara fishing households in Lázaro and Saco da Ribeira. From the 1960s onwards, Caiçaras lost thousands of hectares of land as Ubatuba gained road connections and immigrants took advantage of the lack of legal title and illiteracy of local residents to take ownership of these areas (Adams 2002). The families that could keep land are usually very proud of and attached to it. Currently, land is quite expensive in the Ubatuba area, making it hard for many Caiçara families to buy land, especially close to the coastline and with easy access to the sea.

Moreover, as Idrobo and Johnson (2020) found, owning and renting out a second house is generally perceived to be one of the best opportunities for taking advantage of tourism, as it offers a significant source of income during holidays and the high season. Frequently, it is the income from renting a second house that provides fishers the financial security to maintain fishing activities throughout the year, even when catches are low. This is even more the case for households where women are not involved in paid jobs. Nevertheless, rental agreements, cleaning and preparing the rental house for tourists, and managing the household budget are tasks largely conducted by women. All male individuals who rated their levels of satisfaction with the “land ownership” area as “good” or “excellent” were those that had land within the community. Finally, in Ubatuba, Caiçaras acquire land mainly through inheritance. Consequently, properties are continuously subdivided into smaller parcels from generation to generation to allow for passing down land to sons and daughters. Properties are also subdivided within generations, for constructing second houses to rent to tourists. This might translate into consequences for the future generations of Caiçaras and their access to land title.

##### Job satisfaction

Job satisfaction offered another indication of the high importance placed on fishing for participants (Fig. 7b). Despite income from fishing representing a relatively small percentage of the overall household income (with exceptions for trawler fishers), fishers still dedicate most of their time to fishing activities. This can be explained simply by the fact that the primary sources of revenue for local fishers frequently did not take much of their time (such as managing tenants and seasonal tourist renters and being retired). Of course, another reason is that fishers find great satisfaction in fishing. As an older and a younger male fisher, respectively, explained:

Yeah, it [fishing] is like an addiction for me, if I stay here [on land] I get anxious to go to the sea. I always want to go to the sea. It is a good thing, a good addiction, not a bad one.

To fish is a profession, but it is also a pleasure, it is doing what I like. If I am frustrated, I go to the sea and all changes. I feel good at the sea, fishing. There is no other job that I would rather have.

Probably not coincidentally, the only woman who cited “job satisfaction” (or in her words: “to work with what I love”) as one of the five most important areas for wellbeing was a fisherwoman. Attachment to fishing is also reflected by the high satisfaction levels in the “job satisfaction” area (Fig. 7). Trawler fishers were the only ones who rated levels of satisfaction as “OK,” a reflection of the many restrictions to their fishing activities and consequences of those restrictions for their livelihoods.

Taken in aggregate, the GPGI results provide insights into the primary aspects of Caiçara fisher life in Ubatuba that shape wellbeing. “Family” related areas and “God/faith” show high levels of satisfaction, while “money” and “freedom to fish” show lower satisfaction levels. The data pointed to men being more satisfied with the different chosen wellbeing areas, except satisfaction levels with the domain “God and faith” and “job satisfaction.” The area “job satisfaction,” however, was cited by only one (fisher) woman, who rated it as “excellent” and therefore, this number is not necessarily representative of the women in the study area.

Our assessments revealed important nuances and variations within the experience of research participants regarding the influence of identity on wellbeing. Most obvious from the GPGI data is the distinction in wellbeing by gender identity. For men, fishing identity is prominent and enormously subjectively important to the point where it shapes their spirituality in ways that diverge from that of women. At the same time, however, men whose lives are most dependent on fishing for their livelihood are less satisfied than those with more diverse livelihoods and better social networks. While familial, community, and religious relational identities were important for men and women, emphasis on them was greater for women. That women’s evaluations of these aspects of their wellbeing were generally less positive than men perhaps relates to their greater responsibilities in these spheres of life. Women also have much less access to fishing as an alternative occupation than men, which restricts options beyond their land-based activities.

#### The relational landscape of Caiçara fishers in Lázaro and Saco da Ribeira

**Fig. 8:**
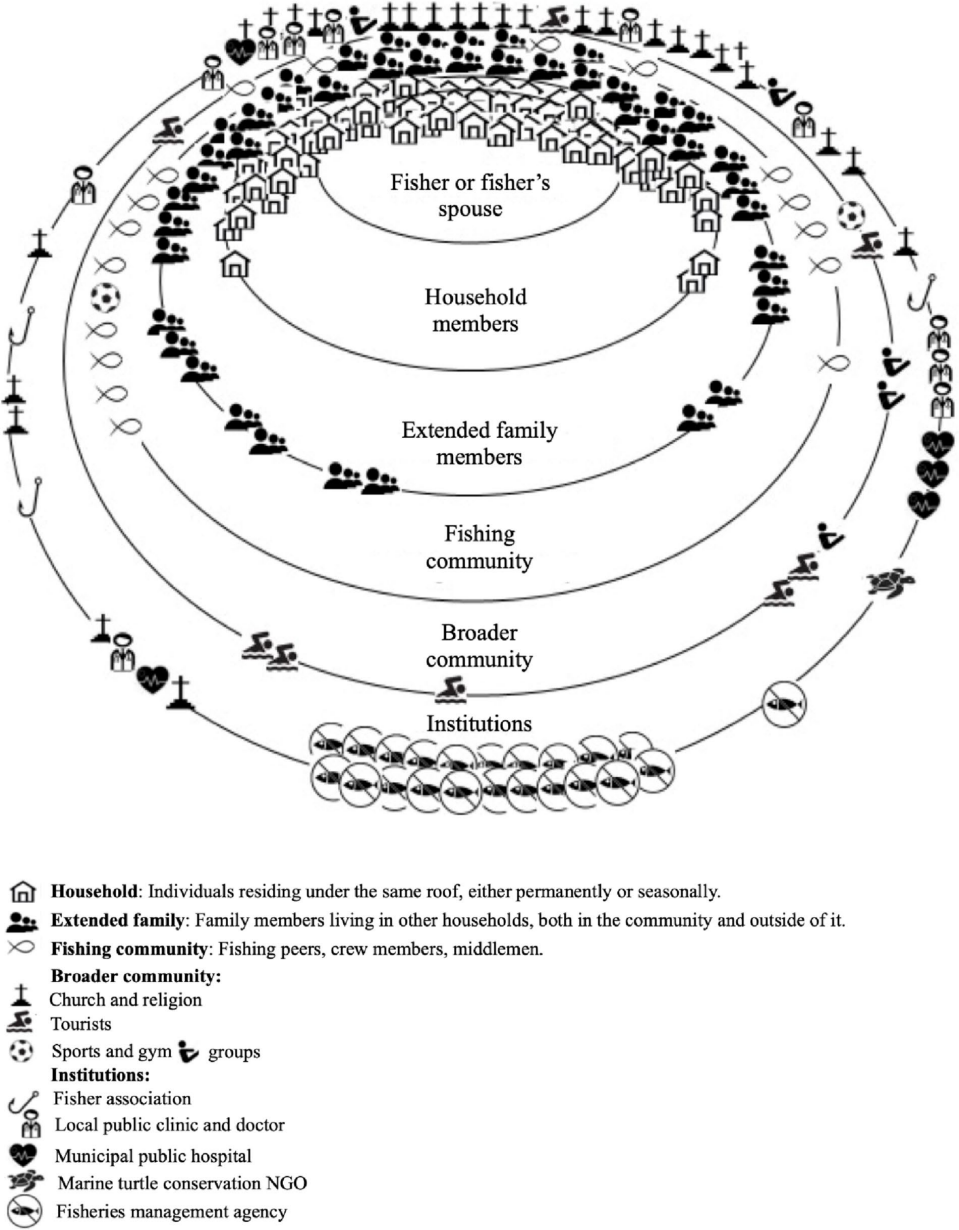
Relational landscape based on the relationship landscape method. Research participants (*n*=30; 18 men and 12 women) identified their most important relationships at several levels. Their satisfaction with each kind of relationship was placed on the corresponding level, with proximity to the top of the circle indicating relative positive satisfaction with the relationship. The innermost circle represents the research participant. The next circle (from inside moving out) refers to their relationship with other household members, the third circle represents their relationships with extended family members, followed by their relationships with the fishing community, the broader community and finally, the outermost circle refers to relationships with institutions

The relational landscape method was applied to participants individually during the semi-structured interviews (18 men and 12 women). It changed the wellbeing focus from subjective to relational by asking research participants to give the relative importance of relationships with different categories of actors. In Fig. 8, the circles represent levels of relationships. Participants could choose to place specific relationships as close to or as far from the inner circle (which represented themselves) as they desired, depending on the degree of proximity they wanted to express. Taken as a whole, the figure gives a qualitative impression of the relative importance of each type of relationship to the research participants. As such, the assessment of importance of different categories of relationship has subjective wellbeing implications.

The relational landscape method revealed the importance of family (including household and extended family members) for Caiçara fishers and their partners. Moreover, data from the youth focus group confirmed that also among the younger generation, strong ties remain centered on the family unit, as siblings and cousins were commonly referred to as the closest relationships, followed by sports groups. At the “fishing community” level, fishing peers were shown to be primary relationships for fishers. Fishing together and sharing boats and gear were common customs for small-scale fishers in the study area. Fishing peers occurred frequently within households, extended families, and between members of traditional extended families in the community. However, there are cases where fishing together transcends family units, particularly for trawler fishers, who frequently rely on crew members, and therefore have a more established working regime and agreement. Relations with tourists also are important interactions at the broader community level, as many have significant dependence on tourism for their livelihoods.

At the institutional level, within the community, churches demonstrate the most substantial weight for Caiçaras from Lázaro and Saco da Ribeira in terms of relational wellbeing. The local clinic and especially relationships with the clinic doctor were cited as the most important relationship between participants and local public services. The Saco da Ribeira Fisher Association, which was inactive until the end of the fieldwork, was cited by only three fishers. This status changed, however, at the time of the validation trip in May 2016 when the organization had been reignited. Follow-up research would be required to understand the association’s effects on relational wellbeing and fishers’ ability to better influence fisheries management and policy. Last, the “relational landscape” indicates an absence of engagement and collaboration between local fishers and fisheries management institutions, as all participants placed relationships with government agencies responsible for fisheries management in the far end of the institutional circle, reflecting the absence of dialog between fishers and managers in the place of study.

Our findings show, in sum, a distinctive social relational landscape in Lázaro and Saco da Ribeira that accentuates tendencies in the GPGI data above. Family, religion, and fishing relationships run strongly through the ways in which people conceive of their wellbeing. As the GPGI data show, however, the intensity of these relational identities varies by particular individual circumstances and history.

### Contextualizing the findings within the social wellbeing approach

**Fig. 9:**
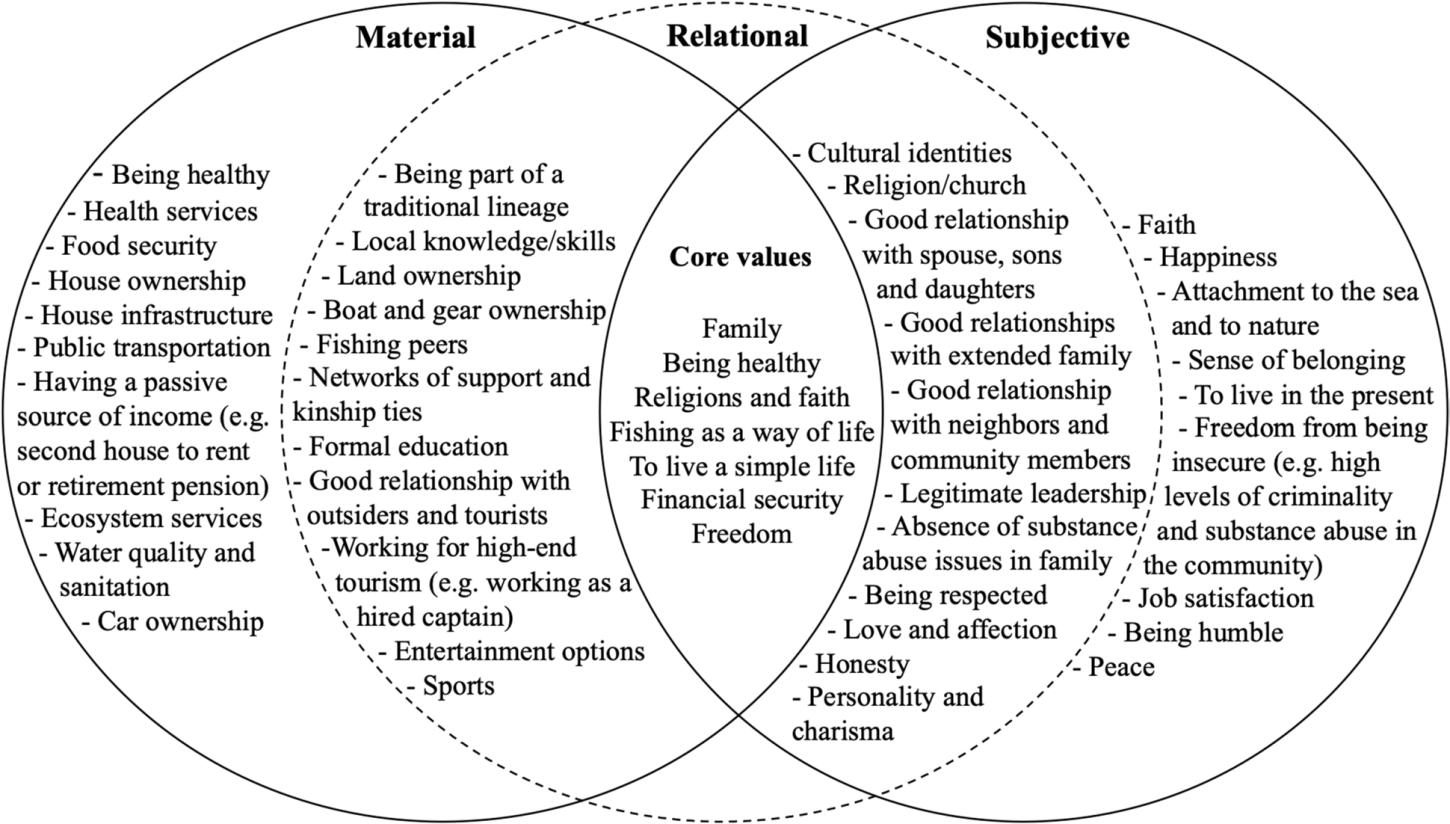
A heuristic representation of the most relevant wellbeing areas of research participants divided into the three analytical dimensions of the social wellbeing approach

We organize the central findings of our study across the three social wellbeing dimensions. In Fig. 9, we distill core values of Caiçara fishers’ identity from the preceding GPGI and relational landscape data. We see these values as referential, not as a set that fits together to constitute a homogenous Caiçara fisher identity. These reference values inform identity and wellbeing for Caiçara fishers in complex and contradictory ways. Figure 9 is specific to this research context in that we have generated it inductively based on data from our study. Nevertheless, the figure may be relevant to studies of other Caiçara communities or fishing communities more generally.

In Fig. 9, instead of each characteristic being exclusively positioned in one of the three wellbeing dimensions, we emphasize tendencies of different wellbeing areas to fit in each segment, while recognizing that many aspects have clear connections to other wellbeing dimensions. The interactions between dimensions of wellbeing illustrated here aim to reinforce this point. We stress the significance of relationships by placing the “relational circle” at the center of the figure and representing its domain with a dashed line. This implies that relations are crucial in defining what wellbeing means for participants, especially through the core values of the embrace of a simple life, fishing as a way of life, and the importance of the family unit, faith, and freedom. We reiterate that the subjective evaluation of social relations and the identities given by those relations vary circumstantially, as we show particularly in relation to gender difference through our GPGI findings.

## Discussion: broader implications for research on wellbeing and governance in small-scale fisheries

Identity is central to the literature on small-scale fisheries and one of the main justifications for research on small-scale fisheries. In this section, we reflect on implications of our research with Caiçara fishers in Ubatuba for this central premise of the small-scale fisheries literature. Social wellbeing as an analytical method complicates notions of small-scale fisher identity through careful attention to social relational context. The social wellbeing method reveals that fisher identity in Lázaro and Saco de Ribeira is cross-cut with Caiçara and other identities and is subject to varying individual capabilities, social positions, and networks. Different strands of identity fit together imperfectly and often conflict. Nonetheless, our research also shows that environmental governance based on exclusionary protected areas has directly threatened material, subjective, and relational dimensions of Caiçara identity and, thus, Caiçara wellbeing in Lázaro and Saco de Ribeira. Whether the Caiçara living in this area can confront this threat effectively depends greatly on whether their sense of shared identity can transcend the many social and identity differences that threaten to fragment it.

### Complementarities and tensions in Caiçara fisher identity

Fishing is central to the economy and identity of the Caiçara population in Lázaro and Saco de Ribeira but fishing is not the sole referent of wellbeing for Caiçara in this place, nor does fishing mean the same thing for all members of the community. We illustrate the importance of Caiçara fisher identity in this section but also the ambiguity of the label with reference particularly to the different view on the question by men and women.

The observation that men and women have different orientations to fishing identity is a long-standing theme in the fisheries literature (Nadel-Klein and Davis 1988) if one that continues to need assertion and research (Weeratunge 2010; Frangoudes and Gerrard 2018). In simplified terms, the distinction in the fisheries literature is between two kinds of freedom. For fishers, primarily male, freedom is the ability to fish independently, often supported by a functionalist logic that fishing requires courage and self-reliance due to the exposed conditions at sea (McGoodwin 1990). In contrast, shore work, primarily by women, is the freedom given by strong webs of social relations — of family and community — that provide support that enable economic success and resilience to the variabilities of fishing livelihoods.

These themes in the fisheries literature are complemented by the terms of the social wellbeing perspective. An important observation in the wellbeing literature is that perceptions of both material and relational wellbeing are influenced by subjective interpretations of what matters for different people to live well (White 2008). Wellbeing researchers working in global south countries have found (Fischer 2014; Narayan et al. 2000) that material resources, health, and family are essential components for living well. On the other hand, lack of freedom and voice, or feelings of “powerlessness,” is commonly experienced by people as factors decreasing wellbeing (Narayan et al. 2000). In the terms of the fisheries literature, subjective weight for fishers is on their freedom to fish, while for the shore crew, subjective emphasis is on the networks of support that provide the relational basis for the resilience of their household and community wellbeing.

The findings of our research in these areas are similar to observations that Britton and Coulthard (2013) make in a study conducted in Northern Ireland using the social wellbeing approach. In their work, as ours, family and health were the most important wellbeing areas. Furthermore, job satisfaction, material security, freedom, and faith were identified as central elements of wellbeing in both studies. Another similarity was that community relations (expressed as “community spirit” in Britton and Coulthard, 2013) was identified as more relevant to women than men in both contexts. While fishers are at sea, their (non-fishing) wives are in the community, and therefore quite frequently build stronger ties with non-fisher community members. In that sense, women are often pillars of support for fishermen and for household social life (McGoodwin 1990). Indeed, in our study, women reported both the categories “Relationships with neighbors and friends” and “God and faith” as more relevant for their relational wellbeing than did men. Whereas the participation of women in fishing related activities, such as pre- and post-fishing activities (see Bennett 2005; FAO Fisheries and Aquaculture Department 2007; Frangoudes 2011; Kawarazuka and Béné 2011) and their central role in domestic and care work (provided for children and seniors) are widely reported (Harper et al. 2013), the social roles of women in the life of fishing communities continue to be less understood (Frangoudes and Gerrard 2018). Importantly, women’s simultaneous responsibilities for domestic work, pre- and post-fishing-related activities, building networks of support, and frequent financial contributions to household income (by engaging in paid jobs) result in costs for their own wellbeing for the sake of the wellbeing of the family unit. This is a trade-off also reported in communities that practise small-scale agriculture (Alston 2006; Gartaula et al. 2017).

The need for independence, and pride, as core components of job satisfaction is a theme well explored in the literature on fisheries (Pollnac and Poggie 2006; Pollnac, Pomeroy, and Harkes 2001). Britton and Coulthard (2013) identified “job satisfaction” as the area of wellbeing that men would most like to change in Ireland due to the impacts of restrictive policies on their fishing activities. The same was reflected in Caiçara fishers’ low levels of satisfaction regarding the “freedom to fish” area. Nevertheless, in our research, fishers tended to associate “job satisfaction” with attachment to fishing activities, which was rated highly, despite all the challenges imposed by policies. This difference might be explained by the fact that most fisher households in Lázaro and Saco da Ribeira did not have fishing as their main source of income, while most fishers in the Northern Ireland community context relied primarily on fishing for their livelihoods (Britton and Coulthard 2013). In fact, the fishers who had lower satisfaction levels with the “job satisfaction” area in our case study were, just like fishers in Northern Ireland, the ones who relied mostly on fishing for income. These findings complicate assertions that fisher subjective wellbeing is primarily about the realization of freedom to fish. It suggests that the very freedom to fish may best be met as part of more plural livelihood strategies that necessitate landward engagements and transcend overly rigid boundaries on men’s and women’s worlds. This recognition of the inter-relation of men’s and women’s worlds then brings the possibility of more fully understanding the social relational challenges that hamper wellbeing and the capacity of the Caiçara population in Lázaro and Saco de Ribeira to engage in governance.

### Trade-offs between wellbeing dimensions: religion, community cohesion, and economic development

Our research revealed tensions and trade-offs between different aspects of wellbeing for participants, such as the influences of religion on individual and households’ networks of support, as well as on the overall community and its cohesion. The relevance of religion for improved subjective wellbeing was evident as there were robust networks of support among members of each of the church groups. These networks were pointed to as essential in times of illness, loss, death, and financial hardship, as well as supporting recovery from substance abuse. Indeed, religion is frequently associated in the literature with networks of support (Ivtzan et al. 2013; Hill and Pargament 2003), not only during life events but also as a protection against substance abuse (Moreira-Almeida, Neto, and Koenig 2006). Alcohol and drug problems are a common challenge faced by fishers worldwide, as researchers have found for decades (McGoodwin 1990; Pollnac and Poggie 2008). As our findings demonstrated, faith and religion are important aspects to be considered for both subjective and relational wellbeing. As they address the costs of addiction, they also have implications for material wellbeing.

Even though our analysis showed that religion and participation in church circles seemed to play a larger role for women, male fishers also declared a robust faith and subjective experiences related to fishing and being at sea. In particular, we found that the sea can be considered a source of spirituality for fishers, as it evokes this grander purpose, such as a deep connection with nature; and gives meaning to their lives, as fishing is their job and joy. Broadening the scope of attachment to nature more generally, the literature on restorative environments emphasizes the importance of nature as a source of relief for individuals attached, in some way, to a specific landscape (Frumkin 2003; Hartig 2007; Kaplan and Kaplan 1989). Different individuals will perceive different landscapes depending on the meanings they attribute to these specific environments, their attachment to them, and their life trajectories in them (Abraham, Sommerhalder, and Abel 2010). As Caiçara fishers explained, the sea and fishing represent more than an income source, they embed a way of life (Trimble and Johnson 2013) that reflects their values, beliefs, and identity.

Broadly, there is substantial research that supports the association of spirituality and religion with mental health and subjective wellbeing (Ivtzan et al. 2013; Hill and Pargament 2003). Yet, there is also vast literature on the negative impacts of religion, which can lead to poor mental health states for individuals, or religious groups, that behave in “judgmental, alienating and exclusive” ways (Williams and Sternthal 2007: 48). Religion, as experienced historically, can also be used to justify exclusion, hatred, and aggression (Williams and Sternthal 2007). In a fisheries context, Lokuge and Munas’ (2018) research in Sri Lanka points to the influence of diverse religious values on fishers’ behavior and decision making, and how religious discourses and ethnic tensions play a critical role in the dynamics around rivalry over access to fishing resources. In Ubatuba, religion did not influence access to resources, but rather created divisions between fishing families belonging to different churches, which we have shown had a negative impact on the cohesion of the broader fishing community.

In this research, religion was shown to act in multi-faceted ways and was characterized by trade-offs. The schisms created by competing religious identities in Ubatuba have implications for the collective agency of Caiçara small-scale fishers. Their capacity to self-organize and engage in governance is challenged when critical networks of support are not at the fishing community level, but rather within church circles (Leite et al. 2019). These denominational orientations have strengthened the systems of support of sub-community groups and increased the subjective wellbeing of many individuals as indicated in the area of faith, and also sense of belonging. At the level of women’s individual wellbeing, religion also posed a trade-off between subjective and relational dimensions. Many women seemed to prioritize their faith — and consequently connection with their church network (subjective and one level of relational wellbeing) at the cost of their more extended community-level relations, including with extended family members who do not share the same beliefs. This finding shows how social wellbeing methodology draws out the social cleavages that complicate possibilities for collective action in small-scale fisheries.

Economic development in Ubatuba has also resulted in many trade-offs that have intersected with the increasing schisms in local faith practices. These trade-offs included improved material wellbeing, food security, access to the health system, and increased incomes, at the cost of weakened community cohesion and loss of social capital due to the more individualistic lifestyles characteristic of modern societies and differences in religious affiliation. As previously mentioned, social challenges, such as substance abuse, marginalization, and also increased crime were also negative effects of economic development in the area, and examples of common and longstanding issues faced by fishing communities worldwide (McGoodwin 1990; Pollnac and Poggie 2008, Leite et al. 2019). In addition to the negative impact of economic development, restrictive environmental policies also affect fishers’ wellbeing by limiting their freedom to fish, as discussed in the next section.

### Social wellbeing as a lens on governance

Freedoms of doing and being what one has reason to value are an essential component of wellbeing (Sen 1999). As we have already made clear, relations with the sea are fundamental to Caiçara fisher perceptions of wellbeing. Yet, here, fisheries governance arrangements and intra-community differences have combined to constrain Caiçaras’ capacity to “lead the kind of lives which [they] have reason to value” (Sen, 1999:14). Participants in our research pointed to lack of freedom and voice or feelings of “powerlessness” as factors that created illbeing. This is a common experience for poor people in developing countries (Narayan et al., 2000). Part of this sense of powerlessness among our research participants relates to the history of loss of land by the Caiçaras of Lázaro and Saco da Ribeira. We found, however, that Caiçara fishers most commonly associate restrictive fisheries management policies with feelings of powerlessness and constrained freedom. Fishers are deeply resentful that their historical use rights to productive fishing grounds have been curtailed and they find state-led governance processes onerous and inconclusive. In this light, fishing illegally in neighboring marine protected areas can be seen as an act of resistance to imposed constraints on Caiçara fisher freedom. Moreover, the reported decreases in community cohesion have had an important impact on fishers’ collective agency and their ability to advocate for better representation in policies that directly affect their ways of life and overall wellbeing. These connections between identity, divisions in the community fabric, relations with external management authorities, and constraints on agency demonstrate that the social wellbeing approach provides an approach to understand fundamental constituents of governance in particular places.

It is important to highlight, though, that these expressions of powerlessness by Caiçara residents of Lázaro and Saco da Ribeira have parallels among all Caiçara communities. A large portion of Caiçara traditional lands have been encompassed by no-take protected areas established over the past 50 years that have imposed restrictions on their livelihoods. In those communities, a movement of Caiçara resistance to fight for their rights in contexts of environmental racism and social exclusion (Adams, 2002) and to engage in participatory management (Bockstael et al. 2016, Araujo et al. 2017) has been seen in the past decades. Such movements have been led often by young adults (but not exclusively) and culminated in the establishment of a National Coordination of Caiçara People in 2014 (Fourth author’s fieldnotes). Even in Lázaro and Saco da Ribeira, the reactivation of the fishing organization — after fieldwork for this research ended — led by a young adult in face of further restriction on fishing gears imposed by the state is a sign that agency as part of Caiçara identity is a latent (or dormant) feature (Leite 2018).

## Conclusions

As with small-scale fisheries in all parts of the world, Caiçara fishers and their families in Ubatuba are seeking to sustain their wellbeing in the face of numerous long- and short-term challenges. Despite these pressures, in 2014–2015^1^, the Caiçaras with whom we interacted expressed a surprisingly resilient view of the wellbeing of themselves and their families. We think that strength of spirit is linked to the identity, and related values, of the people of Lázaro and Saco da Ribeira as fishers and as Caiçaras. It comes from common experiences of work and life in the marine environment, valorization of that particular way of life, and relations with indifferent or even exploitative external actors. The relational ties to community and environment that characterize Caiçara life in Lázaro and Saco da Ribeira are a substrate that maritime anthropology would point to as cultural regularities in life or identity. However, as our social wellbeing analysis makes clear, Caiçara fisher identities are not even, but rather vary by social position, personal history, and patterns of work and belief. This complexity of experience is the counterpoint to claims for the shared cultural distinctiveness of small-scale fisheries. There are commonalities of small-scale fisher experience, yes, but they must be balanced with the recognition of the variability between and also within small-scale fisheries. In Ubatuba, it is particularly Caiçara identity that most strikingly marks local small-scale fisheries as distinctive. But it must be emphasized, Caiçara identity is intersected by, and experienced in conjunction with, other facets of life that social wellbeing so adeptly draws out.

The social wellbeing approach, with its three-dimensions, proved to be a powerful tool for expanding and deepening the analysis of Caiçara fishing families’ quality of life. Beyond material dimensions of wellbeing (i.e., income), and institutional levels of relations with government agencies and other actors, the approach pays careful attention to subjective and relational wellbeing. These are areas normally neglected in livelihood studies, especially at more micro scales such as the individual and household levels (Weeratunge et al. 2014). Even if options exist to fulfill material needs (i.e., access to resources), other important aspects of wellbeing such as values, beliefs, social networks, job satisfaction, and identity can underpin fishers’ behavior and choices (Armitage et al. 2012).

In this sense, wellbeing is perceived as more than a desirable state of being; it also offers a way to understand fishers’ motivations and worldviews (Britton and Coulthard 2013). Social wellbeing analysis thus has the promise to provide a stronger basis for engagement and understanding between external governance actors and members of small-scale fishing communities, as it reveals aspects of wellbeing such as values, identity, and social relations that are constantly informing fishers’ behaviors, and consequently affecting the successes or failures of fisheries governance. At the point where we conducted our research, the future of Caiçara fishers of Lázaro and Saco da Ribeira was uncertain due to restrictive fishing policies threatening small-scale fishing as a way of life and fisher ability to mobilize to assert, in particular, rights to coastal and maritime spaces. The years since 2015 have been politically and economically tumultuous in Brazil in ways that make the questions we have asked in this paper about change, identity, wellbeing, and capacity for collective action even more urgent.

Our findings show, foremost, that the social wellbeing approach can highlight important collective tendencies in particular small-scale fisheries, but also social rifts that cut across shared identity. Awareness of these characteristics can build understanding by fisheries managers and other external actors, while also pointing to potential challenges for coordination of action and areas where work at community building can perhaps be most effective. Specifically, for the small-scale Caiçara fishers of Ubatuba, fishing clearly has more than just economic value; it is also a source of pride and satisfaction. Nevertheless, the subjective satisfactions associated with fishing have to be juxtaposed with elements of life that decrease wellbeing or reduce the capacity of fishers and their families to live a life free of fear and anxiety. In this study, conflicts with the enforcement agents of fisheries policies, threats to community cohesion, and lack of voice and participation in decision-making are all chronic areas of vulnerability that threaten wellbeing. Our social wellbeing derived observations are fundamental for the development and implementation of policies aimed at helping the small-scale fishers of Ubatuba achieve meaningful and dignified lives in a broader social environment in which they are respected.

## Footnotes

1 Recent tumultuous political and economic changes in Brazil and the COVID-19 crisis have had a major impact on Ubatuba. As we have not been able to conduct systematic follow-up research since the data was collected, however, we leave an account of the impact of these factors until follow-up research can be conducted
